# DeepGraphGO: graph neural network for large-scale, multispecies protein function prediction

**DOI:** 10.1093/bioinformatics/btab270

**Published:** 2021-07-12

**Authors:** Ronghui You, Shuwei Yao, Hiroshi Mamitsuka, Shanfeng Zhu

**Affiliations:** School of Computer Science, Fudan University, Shanghai 200433, China; School of Computer Science, Fudan University, Shanghai 200433, China; Bioinformatics Center, Institute for Chemical Research, Kyoto University, Uji, Kyoto Prefecture 611-0011, Japan; Department of Computer Science, Aalto University, Espoo, Finland; Institute of Science and Technology for Brain-Inspired Intelligence and Shanghai Institute of Artificial Intelligence Algorithms, Fudan University, Shanghai 200433, China; Ministry of Education, Key Laboratory of Computational Neuroscience and Brain-Inspired Intelligence (Fudan University), Shanghai 200433, China; Shanghai Key Lab of Intelligent Information Processing, Fudan University, Shanghai 200433, China; MOE Frontiers Center for Brain Science, Fudan University, Shanghai 200433, China; Zhangjiang Fudan International Innovation Center, Shanghai 200433, China

## Abstract

**Motivation:**

Automated function prediction (AFP) of proteins is a large-scale multi-label classification problem. Two limitations of most network-based methods for AFP are (i) a single model must be trained for each species and (ii) protein sequence information is totally ignored. These limitations cause weaker performance than sequence-based methods. Thus, the challenge is how to develop a powerful network-based method for AFP to overcome these limitations.

**Results:**

We propose DeepGraphGO, an end-to-end, multispecies graph neural network-based method for AFP, which makes the most of both protein sequence and high-order protein network information. Our multispecies strategy allows one single model to be trained for all species, indicating a larger number of training samples than existing methods. Extensive experiments with a large-scale dataset show that DeepGraphGO outperforms a number of competing state-of-the-art methods significantly, including DeepGOPlus and three representative network-based methods: GeneMANIA, deepNF and clusDCA. We further confirm the effectiveness of our multispecies strategy and the advantage of DeepGraphGO over so-called difficult proteins. Finally, we integrate DeepGraphGO into the state-of-the-art ensemble method, NetGO, as a component and achieve a further performance improvement.

**Availability and implementation:**

https://github.com/yourh/DeepGraphGO.

**Supplementary information:**

Supplementary data are available at *Bioinformatics* online.

## 1 Introduction

Proteins are building blocks of life, playing many crucial roles within organisms, such as catalyzing chemical reactions, coordinating signal pathway and providing structural support to cells (Weaver, 2011). In order to elucidate the mechanism of life, it is important to identify protein/gene functions, which are now standarized by Gene Ontology (GO) (Ashburner *et al.*, 2000). The GO covers three biological domains: molecular function ontology (MFO), biological process ontology (BPO) and cellular component ontology (CCO) with over 44 000 concepts (January 2021). The number of known protein sequences increases rapidly due to the development of gene sequencing technologies. Until Jan. 2021, there are more than 200 million proteins in UniProKB (UniProt Consortium, 2019). However, only <0.1% proteins have experimental GO annotations due to the high cost of biochemical experiments. Therefore, to reduce this huge gap, developing an effective and efficient automatic protein function prediction (AFP) method is of great significance (Radivojac *et al.*, 2013).

For assessing the performance of large-scale AFP methods, Function Special Interest Group (Function-SIG) of International Society for Computational Biology (ISCB) has organized a community challenge, the Critical Assessment of protein Function Annotation algorithms (CAFA) (Jiang *et al.*, 2016; Radivojac *et al.*, 2013; Zhou *et al.*, 2019). CAFA has been held four times so far: CAFA1 in 2010–2011, CAFA2 in 2013–2014, CAFA3 in 2015–2016 and CAFA4 in 2019–2020 (prediction results of CAFA4 are still under evaluation). In both CAFA3 and CAFA4, the organizers provided a large number number of protein sequences (around 100 000) to the participants, who have to submit the predictions of protein functions (GO term associations) before the deadline (T0). For building the benchmark data, then the organizers collect proteins with experimental annotations by a few months later (T1, 10 months later in CAFA3). The benchmark data consists of two types of proteins: *no-knowledge* and *limited-knowledge* proteins (Zhou *et al.*, 2019). Without any experimental annotations before T0, *no-knowledge* proteins receive at least one experimental annotation between T0 and T1. On the other hand, *limited-knowledge* proteins have partial prior experimental annotations before T0 in one or two domains other than the target domain, where the first experimental annotation was obtained between T0 and T1. Since currently more than 99.9% proteins have no experimental annotations, we focus on AFP for *no-knowledge* proteins in this study.

One protein can be associated with multiple GO terms. By regrading each GO term as a label and each protein as an instance, AFP can be deemed as a large-scale, multi-label problem. This is a challenging task from both sides of label (GO) and instance (protein). For the label side, there are more than 44 000 GO terms, where GO is a directed acyclic graph (DAG), meaning that for one protein annotated by one GO term, all ancestor GO terms in GO can be also assigned. In fact, one human protein is currently associated with 47 GO terms on average, according to Gene Ontology Annotation (GOA) Database (Dec 2020) (Huntley *et al.*, 2015). For the instance side, we can consider all kinds of protein information to improve the accuracy of AFP.

Recently we developed a sequence-based AFP method, GOLabeler (You *et al.*, 2018), which achieved the first place in CAFA3 on *no-knowledge* benchmark in terms of F${}_{\text{max}}$ in all three GO domains. GOLabeler utilizes learning to rank (LTR) to integrate multiple types of sequence information, such as sequence homology, protein domain and family, to rank the candidate GO terms for a given protein. However, sequence information is insufficient to characterize protein functions. A promising idea to improve AFP is that proteins connected in a protein network (e.g. protein-protein interaction or metabolic network) like to share the same functions (Oliver, 2000; Schwikowski *et al.*, 2000). In light of this perspective, we have developed NetGO (You *et al.*, 2019), keeping the LTR framework of GOLabeler, to improve the performance of GOLabeler by massive network information in STRING (Szklarczyk *et al.*, 2019). As a result, NetGO achieved the state-of-the-art performance, while LTR, an ensemble approach of many component methods, is computationally intensive. More importantly, in NetGO, the component method on networks considers only neighbors of a test protein (i.e. low-order information) in given networks, meaning that high-order information in protein networks are ignored.

We propose DeepGraphGO, a semi-supervised, deep learning method, which takes the advantages of both protein sequence and network information through graph neural network (GNN) (Kipf and Welling, 2016). DeepGraphGO has the following three notable features: (i) **InterPro for representation vector**: The input of representation vectors (of nodes/proteins), trained by GNN, is generated from InterPro (Mitchell *et al.*, 2019), a protein domain and family database. It combines 14 different databases, such as Pfam (Finn *et al.*, 2016), SUPERFAMILY (Oates *et al.*, 2015), CATH-Gene3D (Lewis *et al.*, 2018) and CDD (Marchler-Bauer *et al.*, 2017), which provides many types of functional information, such as family, domain and motifs. The features extracted from InterPro were successfully used in GOLabeler and NetGO as well. (ii) **Multiple graph convolutional neural (GCN) layers**: GNN has been developed for various tasks, such as node embedding, link prediction, node classification and graph classification (Zhou *et al.*, 2018). Graph convolutional network (GCN) is a typical GNN. It can obtain a representation vector of each node by a graph convolutional layer (GCN layer), which aggregates representations of neighboring nodes. Multiple GCN layers allow to capture high-order information among nodes (proteins). (iii) **Multispecies strategy**: We used proteins of all species for training only one single model, which we call *multispecies strategy*. Compared with previous work focusing on single species, it can make use of more data to achieve better performance, especially for the species that are sparsely annotated.

We thoroughly validated the performance of DeepGraphGO through comprehensive experiments on large-scale datasets under the CAFA settings. We compared DeepGraphGO with a number of methods, including DeepGOPlus (Kulmanov and Hoehndorf, 2020), a state-of-the-art deep learning-based method for AFP, and three most important components of the latest ensemble method, NetGO: BLAST-KNN, Net-KNN and LR-InterPro. Experimental results demonstrate that DeepGraphGO outperformed all competing methods in F${}_{\text{max}}$ and AUPR for all three domains of GO. We confirmed that our multispecies strategy of using all species for one single model is effective: DeepGraphGO outperformed DeepGraphGO${}_{\text{sp}}$, which was trained by only proteins of a specific species. Also, even DeepGraphGO${}_{∼sp}$, which was trained by proteins of all other species except the specific species, outperformed DeepGraphGO${}_{\text{sp}}$. This indicates that using other species is useful, confirming our multispecies strategy. All these results prove the effectiveness and efficiency of DeepGraphGO. Finally, we integrate DeepGraphGO into NetGO as a component to generate a model, called DeepGraphGo-LTR. It outperformed the two state-of-the-art ensemble methods, GOLabeler and NetGO in all three domains of GO in our experiments, showing the possibility of improving the predictive performance of AFP further.

## 2 Related work

There are a large number of studies for AFP (Zhou *et al.*, 2019), while the network-based methods are most related.

There are three well established network-based methods for AFP: GeneMANIA (Mostafavi *et al.*, 2008), Mashup (Cho *et al.*, 2016) and clusDCA (Wang *et al.*, 2015). GeneMANIA integrates multiple protein networks into one network, over which labels (GO terms) are propagated for prediction. Mashup learns the embeddings of proteins by using a method called diffusion component analysis (DCA) over a given network, and these embeddings are used for prediction. ClusDCA also uses DCA, while embeddings of proteins and GO terms are trained from protein networks and the DAG of GO, respectively. These three methods have two clear drawbacks: (i) the prediction model of each species is trained independently. (ii) sequence information is completely ignored. Thus a more recent method, ProSNet integrates both sequence homology and molecular network to improve the performance of AFP (Wang *et al.*, 2017). However, the complexity of constructing and training a network in ProsNet is extremely high, which makes it infeasible to incorporate a dozen of species at the same time. Also, the performance of ProSNet was examined by cross-validation, while separating test data from training data is not clear in a network, casting a doubt as to whether a protein in test data is new for training data. Note that the validation setting of CAFA (which will be used in our experiments) uses *no-knowledge* proteins, which can clearly avoid the above doubt of cross-validation in network data.

The cutting-edge deep learning-based methods for AFP also use protein networks as input. By running a deep graph autoencoder on a given network, deepNF (Gligorijević *et al.*, 2018) learns representation vectors for proteins which are used for building a support vector machine (SVM) classifier for each GO term. Graph2GO (Fan *et al.*, 2020) takes a similar procedure, while various information, including protein sequences, subcellular location and protein domains as well as protein networks are all used to generate representation vectors. A drawback of both deepNF and Graph2GO is that training and testing must be done for each species. DeepGO (Kulmanov *et al.*, 2018) generates representation vectors from both amino acid sequences and protein networks, while the high computational burden of DeepGO limits the label size, like only 2000 out of all more than 44 000 GO terms being a predictable limitation. DeepGOPlus (Kulmanov and Hoehndorf, 2020) is a simpler model to reduce the high computational complexity of DeepGO, combining two submodels, a neural network called DeepGOCNN and a k-nearest neighbor called DiamondScore, in which protein similarity is computed by the Diamond tool (Buchfink *et al.*, 2015). However, empirically both DeepGO and DeepGOCNN provide only lower performances than even DiamondScore, for MFO and BPO (Kulmanov and Hoehndorf, 2020).

Recently, several methods using GNN for AFP have been proposed (Gligorijevic *et al.*, 2019; Ioannidis *et al.*, 2019; Zhou *et al.*, 2020). DeepFRI (Gligorijevic *et al.*, 2019) is a GNN-based method for AFP, which uses LSTM (long short-term memory) to extract residue level features of protein sequence and GCN layers for learning complex structure to function relationships. DeepGOA (Zhou *et al.*, 2020) is another method using GNN for AFP. DeepGOA encodes protein sequence by CNN as DeepGOCNN and obtains a semantic representation of each GO term by GCN on the DAG of GO. Both DeepFRI and DeepGOA do not use PPI network information, and can only deal with a small number of GO terms (around 4000 out of more than 44 000) due to their high computational complexity.

## 3 Materials and methods

### 3.1 Overview

**Fig. 1.**
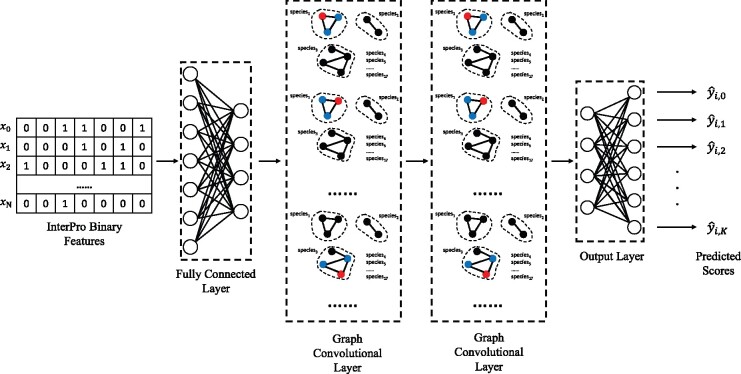
A schematic procedure of DeepGraphGO. The input is *N* binary feature vectors (with the size of *m*) obtained from InterPro. The size of the input vectors is reduced by the input (fully connected) layer [For example, *m* (originally seven) is reduced to four] to generate dense vectors, which are used as the initial value of representation vectors of the subsequent convolutional (GCN) layer. The GCN layer accepts protein networks of all species (due to our multispecies strategy), and the representation vector of each node (in red) is updated by the representation vectors of the connected nodes (in blue). This process is repeated (twice) to capture the neighboring information, eventually high-order information in the given network. Finally the output layer outputs the prediction scores of *K* GO terms for each protein by using a fully connected layer with the input of representation vectors trained by the GCN layers

Figure 1 shows a schematic procedure of DeepGraphGO, which has two inputs: (i) graph *G* (protein network) with *N* nodes (proteins) or weighted adjacency matrix $A\in R^{N\times N}$ (edge weights range between 0 and 1). (ii) *N* binary feature vectors, generated by InterProScan (Jones *et al.*, 2014) for *N* proteins based on InterPro, where each element shows the presence/absence of a protein domain/family/motif. The procedure has three steps: (i) Input (fully connected) layer: the binary feature vector of each protein is transformed into a non-binary vector, to be used as the initial representation vector. (ii) Graph convolutional (GCN) layer: updates the representation vector of each node (protein) to capture high-order information through graph edges, by renewing the vector using those of neighboring nodes. (iii) Output (fully connected) layer: predicts scores of GO terms for each protein.

### 3.2 Input layer

For protein *p_i_*, we use InterProScan to generate a binary feature vector $x_{i}\in {\left\{0,1\right\}}^{m}$, where *m* is the number of signatures (domains and families in InterPro) related to at least one of *N* proteins in *G*, and the *j*th element of $x_{i},\;x_{i,j}$, indicates if the *j*th signature belongs to *p_i_*. We use a fully connected layer to obtain a low-dimensional representation vector $h_{i}^{\left(0\right)}\in {\mathbb{R}}^{d}$ from $x_{i}$ as follows:

(1) $$h_{i}^{\left(0\right)}=f(W^{\left(0\right)}x_{i}+b^{\left(0\right)}),$$

where $W^{\left(0\right)}\in {\mathbb{R}}^{m\times d}$ and $b^{\left(0\right)}\in {\mathbb{R}}^{d}$ are the weight and bias of the fully connected layer, respectively, and *f* is a non-linear activation function. We generate initial representation matrix $H^{0}\in R^{N\times d}$ by concatenating the obtained low-dimensional representation of all *N* proteins.

### 3.3 GCN layer

Following (Kipf and Welling, 2016), at the *l*th GCN layer (1≤l≤M), representation matrix $H^{\left(l\right)}\in {\mathbb{R}}^{N\times d}$ can be updated with residual connection (He *et al.*, 2016) as follows:

(2) $$H^{\left(l\right)}=f({\tilde{D}}^{-\frac{1}{2}}\tilde{A}{\tilde{D}}^{-\frac{1}{2}}H^{\left(l-1\right)}W^{\left(l\right)}+b^{\left(l\right)})+H^{\left(l-1\right)},$$

where $\tilde{A}=A+I$, *I* is the identity matrix with the size of *N*, $\tilde{D}$ is the degree matrix of $\tilde{A}$ (${\tilde{D}}_{ii}=\sum_{j}{\tilde{A}}_{ij}$) and $W^{\left(l\right)}\in {\mathbb{R}}^{d\times d}$ and $b^{\left(l\right)}\in {\mathbb{R}}^{d}$ are the weight and bias, respectively. Continuous *M* GCN layers can capture high-order [*M* (and less)-order] information of each node.

### 3.4 Output layer and loss function

For the *i*th protein and the *j*th GO term, prediction score ${\hat{y}}_{ij}$ can be computed by the output (fully connected) layer as follow:

(3) $${\hat{y}}_{ij}=\sigma (w_{j}^{\left(o\right)}h_{i}+b_{j}^{\left(o\right)}),$$

where $w_{j}^{\left(o\right)}\in {\mathbb{R}}^{d}$ and $b_{j}^{\left(o\right)}\in \mathbb{R}$ are the weight and bias for the *j*th GO term, respective and *σ* is the sigmoid function. We use the binary cross-entropy as loss function *J*:

(4) $$J=-\frac{1}{NK}\sum_{i=1}^{N}\sum_{j=1}^{K}y_{ij}\;\text{log}({\hat{y}}_{ij})+(1-y_{ij})\log(1-{\hat{y}}_{ij}),$$

where *K* denotes the number of GO terms and $y_{ij}\in \{0,1\}$ is the ground truth. The loss function is computed from only known proteins (nodes with the ground truth in *G*), which means semisupervised learning.

### 3.5 Training setting

We use mini-batch training, with which GraphSAGE shows a better generalized performance (Hamilton *et al.*, 2017), instead of full-batch training in (Kipf and Welling, 2016). Practically, to keep moderate computational complexity, we selected edges with *k* largest weights for each node, instead of sampling neighbors (which is done by GraphSAGE). Note that training uses data of all species, i.e. multispecies strategy.

## 4 Experiments

### 4.1 Datasets

We collected data following the standard CAFA protocol (Jiang *et al.*, 2016; Radivojac *et al.*, 2013; Zhou *et al.*, 2019):

1. Protein sequences: We downloaded protein sequences from UniProt (https://www.uniprot.org/downloads) (UniProt Consortium, 2019).
Protein networks: We used version 11.0 of STRING (https://string-db.org/) (Szklarczyk *et al.*, 2019). This database covers around 24.6 million proteins from 5090 organisms with more than two billion interactions in total, which was generated before T0 (Jan. 2019).
GO terms: We downloaded from SwissProt1 (Boutet *et al.*, 2016), GOA (http://www.ebi.ac.uk/GOA) (Huntley *et al.*, 2015) and GO (http://geneontology.org/page/download-annotations) (Ashburner *et al.*, 2000) in January 2020. We extracted all experimental annotations in: ‘IDA’, ‘IPI’, ‘EXP’, ‘IGI’, ‘IMP’, ‘IEP’, ‘IC’ or ‘TA’. All are combined to generate an annotation dataset.

We then generated training, validation and testing sets by time stamps when proteins were annotated:

1. Training: All data experimentally annotated before Jan. 2018.
2. Validation: All *no-knowledge* proteins experimentally annotated from January to December 2018.
3. Testing: All *no-knowledge* proteins experimentally annotated from January 2019 to January 2020.

For validation and testing sets, we used the same 17 target species as CAFA4. Table 1 shows the statistics of the training, validation and testing sets. Note that DeepGraphGO was trained by proteins in 17 target species appearing in both the training set and STRING, while competing methods (unless using protein networks as input) were trained by all proteins in the training set.

**Table 1.** Data statistics (# proteins) on species with more than 10 proteins in every domain of GO

	Train			Valid			Test		
	MFO	BPO	CCO	MFO	BPO	CCO	MFO	BPO	CCO
HUMAN (9606)	9208	12 095	18 842	86	138	137	41	87	767
MOUSE (10090)	6138	9927	8482	103	299	228	65	156	130
ARATH (3702)	5108	9887	6973	69	166	93	44	100	56
RAT (10116)	5008	8444	9509	86	201	107	97	145	128
DROME (7227)	4312	5412	4912	27	101	140	16	30	25
All species (not only the above)	51 549	85 104	76 098	490	1570	923	426	925	1224
Data used by DeepGraphGO	35 092	54 276	48 093	490	1570	923	426	925	1224
Percentage	68.1%	63.8%	63.2%	100%	100%	100%	100%	100%	100%

### 4.2 Competing methods

Competing methods were used for two types of evaluation manners: protein-centric (The ‘protein-centric’ evaluates GO terms annotated to each protein, while the ‘GO-term centric’ is reverse. The ‘pair centric’ evaluates ‘protein-GO term’ pairs.) and GO term-centric. For protein-centric, DeepGOCNN, DeepGOPlus and three most important components of NetGO: BLAST-KNN, Net-KNN and LR-InterPro. For GO term-centric, three most representative network-based methods: DeepNF, clusDCA and GeneMANIA. These three methods have one model for each species, and each model is trained independently. We explain BLAST-KNN, Net-KNN and LR-InterPro below, while all other competing methods were introduced in Section 2.

#### 4.2.1 BLAST-KNN

The idea is that similar proteins may have similar protein functions. We run BLAST over all proteins in the training set to obtain set *Z_i_* of proteins which are homologous to protein *p_i_* (using a cut-off *e*-value of 0.001 in our experiments). Then score $S_{B}(p_{i},GO_{j})$ between protein *p_i_* and GO term *GO_j_* can be computed as follows:

(5) $$S_{B}(p_{i},GO_{j})=\frac{\sum_{p_{k}\in Z_{i}}I(p_{k},GO_{j})\times B(p_{i},p_{k})}{\sum_{p_{k}\in S_{p_{i}}}B(p_{i},p_{k})},$$

where $B(p_{i},p_{k})$ is the similarity score (bit-score) between *p_i_* and *p_k_* by BLAST and $I(p_{k},GO_{j})$ is a binary indicator: 1 if *GO_j_* belongs to protein *p_k_*; otherwise zero.

#### 4.2.2 Net-KNN

Similarly score $S_{N}(p_{i},GO_{j})$ between protein *p_i_* and GO term *GO_j_* can be computed as follows:

(6) $$S_{N}(p_{i},GO_{j})=\frac{\sum_{p_{k}\in V}I(p_{i},GO_{j})\times \omega (p_{i},p_{k})}{\sum_{p}\omega (p_{i},p_{k})},$$

where *V* is all nodes (proteins) in graph *G* and $\omega (p_{i},p_{k})$ is the weight of the edge between *p_i_* and *p_k_*. In testing, if a given protein $p\prime_{i}$ is not in STRING, the score of protein *p_i_* (in STRING) which is most homologous to $p\prime_{i}$ is used as the prediction score of $p\prime_{i}$.

#### 4.2.3 LR-InterPro

For each GO term, logistic regression (LR) is trained using the binary feature vector (obtained by InsterProScan) which is the same as the input of DeepGraphGO. The trained LR is used for prediction.

### 4.3 Experimental settings

We trained DeepGraphGO for MFO, BPO and CCO separately. We used two GCN layers [In preliminary experiments, we examined deeper GCN layers, while the performance was not highly improved regardless of the dramatically increase of computational cost. Then we set *M* = 2 (see the supplementary material for the details).]. That is, *M *=* *2. The batch size and epoch number were 40 and 10, respectively. We used Adam optimizer (Kingma and Ba, 2014) with the learning rate of 1e-3. We used ReLU (Arora *et al.*, 2016) for activation function *f*. To avoid overfitting, we used dropout (Hinton *et al.*, 2012) after each GCN layer with the drop rate of 0.5. Also, to reduce the computational cost, we used only 30 edges with the largest weights for each node in protein networks, i.e. *k *=* *30. All these hyperparameters were selected by using the validation set. Following NetGO, if a given protein $p\prime_{i}$ was not in STRING, we used the score of protein *p_i_*, which is most homologous (highest bit-score by BLAST) to $p\prime_{i}$ as the prediction score of $p\prime_{i}$. In practice, we trained three models with different initial weights and averaged over the three prediction scores to obtain the final prediction.

For the competing methods, we downloaded their implementations from the original authors’ websites. We trained these methods using our training data and tuned the parameters using the validation data. As GeneMANIA, clusDCA and deepNF utilized multiple networks as input, all 7 types of networks in STRING were used, which include neighborhood, fusion, co-occurrence, co-expression, experiment, database and text mining. For DeepGraphGO, an integrated network from above 7 types provided by STRING was used.

### 4.4 Performance evaluation metrics

We used three evaluation metrics: F${}_{\text{max}}$, AUPR (Area Under the Precision-Recall curve) and M-AUPR. F${}_{\text{max}}$ is protein-centric, which has been used in CAFA as the main evaluation metric (Jiang *et al.*, 2016). AUPR is pair-centric and widely used for performance evaluation of multi-label classification including AFP (Kulmanov and Hoehndorf, 2020; You *et al.*, 2018, 2019). M-AUPR is GO term-centric, being widely used by network-based methods (Gligorijević *et al.*, 2018; Mostafavi *et al.*, 2008; Wang *et al.*, 2015). Specifically, F${}_{\text{max}}$ is defined as follow:

$$F_{\max}=\underset{\tau }{\max}\left\{\frac{2\cdot \text{pr}(\tau )\cdot \text{rc}(\tau )}{\text{pr}(\tau )+\text{rc}(\tau )}\right\},$$

where pr(*τ*) and rc(*τ*) are so-called *precision* and *recall*, respectively, obtained at some cut-off value, *τ*, defined as follows:

$$\begin{array}{c} \text{pr}(\tau )=\frac{1}{h(\tau )}\sum_{j=1}^{h(\tau )}\frac{\sum_{i}1(S(G_{i},P_{j})\geq \tau )\cdot I(G_{i},P_{j})}{\sum_{i}1(S(G_{i},P_{j})\geq \tau )},\\ \text{rc}(\tau )=\frac{1}{N_{T}}\sum_{j=1}^{N_{T}}\frac{\sum_{i}1(S(G_{i},P_{j})\geq \tau )\cdot I(G_{i},P_{j})}{\sum_{i}I(G_{i},P_{j})}, \end{array}$$

where h(τ) is the number of proteins with the score no smaller than *τ* for at least one GO term, and 1(·) is 1 if the input is true; otherwise zero. For F${}_{\text{max}}$ and AUPR, given a testing set, we first obtain the prediction score of each protein-GO term pair. All protein-GO term pairs are then sorted by these prediction scores. Finally, the performance was evaluated by F${}_{\text{max}}$ and AUPR. On the other hand, for M-AURP, we averaged AUPR on each GO term appearing more than twice in a given testing set, where the test proteins are ranked by the prediction scores with respect to each GO term.

### 4.5 Results

In tables of experimental results, the best and second best performance values are highlighted in bold face and underlined, respectively.

#### 4.5.1 Comparison with competing methods over all test proteins

Table 2 shows the performance comparison of DeepGraphGO and all competing methods: BLAST-KNN, LR-InterPro, Net-KNN, DeepGOCNN and DeepGOPlus. We have four main findings: (i) DeepGraphGO achieved the best performance of both F${}_{\text{max}}$ and AUPR in all three domains, especially for BPO and CCO. For example, DeepGraphGO achieved the highest F${}_{\text{max}}$ of 0.327 in BPO, which was 7.2% and 12.8% improvements over Net-KNN (0.305) and DeepGOPlus (0.290), respectively. This result indicates that DeepGraphGO made the most of information of both protein sequences and networks by using graph neural network. (ii) LR-InterPro achieved the second best performance in MFO, which outperformed both BLAST-KNN and DeepGOPlus. LR-InterPro utilized protein domain, family and motif information extracted from InterPro, while BLAST-KNN and DeepGOPlus used only the sequence homology information in BLAST and DIAMOND, respectively. This suggests that protein domain and family information might be more important than sequence homology for function prediction in MFO. (iii) Net-KNN achieved the second best performance in BPO (F${}_{\text{max}}$ and AUPR). This is consistent with widely accepted hypothesis that proteins interacting (connected) in the same network tend to participate in the same biological process. (iv) The sequence-based deep learning method, DeepGOCNN, did not perform well in all three GO domains. This result indicates that encoding protein sequences by a simple one dimensional convolutional neural network is hard to (extract and) capture the most helpful information for AFP.

**Table 2.** Performance comparison of DeepGraphGO and competing methods

Method	F${}_{\text{max}}$			AUPR		
	MFO	BPO	CCO	MFO	BPO	CCO
BLAST-KNN	0.590	0.274	0.650	0.455	0.113	0.570
LR-InterPro	0.617	0.278	0.661	0.530	0.144	0.672
Net-KNN	0.426	0.305	0.667	0.276	0.157	0.641
DeepGOCNN	0.434	0.248	0.632	0.306	0.101	0.573
DeepGOPlus	0.593	0.290	0.672	0.398	0.108	0.595
DeepGraphGO	**0.623**	**0.327**	**0.692**	**0.543**	**0.194**	**0.695**

To check the robustness of improvement by DeepGraphGO, we conducted bootstrap with replacement 100 times, to generate 100 testing sets. We then ran paired t-test over 100 trials to examine the statistical significance on performance improvement between DeepGraphGO and competing methods. The results show that the performance improvements by DeepGraphGO over competing methods were all statistically significant (see Supplementary Materials on the details).

In addition, all methods perform much worse in the BPO category compared to MFO and CCO. This is consistent with the results of CAFA, which could be attributed to the following factors(Jiang *et al.*, 2016; Zhou *et al.*, 2019): (i) BPO has much more GO terms and higher depths than MFO and CCO; (ii) the BPO terms are considered to be more abstract in nature than MFO and CCO terms; (iii) BPO may have complicated annotation status such as the annotation depth of benchmark proteins and various annotation biases.

#### 4.5.2 Performance comparison over proteins in STRING and those homologous to proteins in STRING

To further check the usage of network information in DeepGraphGO, we divided the testing proteins into three subsets: (i) STRI: proteins in STRING, (ii) HOMO: proteins being not in STRING but homologous to proteins in STRING and (iii) NONE: all other proteins. Table 3 shows the number of proteins of these three subsets. We note that proteins in NONE occupy only around 2% of all proteins, although DeepGraphGO is unable to annotate these proteins in NONE (e.g. B2CXA1 and B3H4Y2 in testing data). We note that proteins in NONE occupy only around 2% of all proteins, although DeepGraphGO is unable to annotate these proteins in NONE. Table 4 reports the performance of DeepGraphGO and competing methods on proteins in STRI and HOMO. DeepGraphGO achieved the best performance under all settings, except only one case (AUPR of MFO) for HOMO. Meanwhile, in both cases of STRI and HOMO, LR-InterPro is mainly the second best method for MFO, while Net-KNN is the second best method for BPO and CCO. For example, over STRI proteins, DeepGraphGO achieved the highest F${}_{\text{max}}$ of 0.642 and 0.348 in MFO and BPO, respectively. Subsequently, LR-InterPro achieved the second highest F${}_{\text{max}}$ of 0.630 in MFO, while Net-KNN achieved the second highest F${}_{\text{max}}$ of 0.314 in BPO. In spite of the similar tendency, the degree of improvements by DeepGraphGO over competing methods is much higher in STRI proteins than HOMO proteins. For instance, DeepGraphGO achieved the 10.8% (0.348 versus 0.314) improvement over Net-KNN in terms of F${}_{\text{max}}$ in BPO over STRI proteins, while the improvement over HOMO proteins was only 2% (0.306 versus 0.300). All these results suggest that DeepGaphGO could improve the AFP performance of both STRI and HOMO proteins, particularly STRI proteins because of these proteins appearing in STRING.

**Table 3.** Statistics of subsets STRI and HOMO

	MFO	BPO	CCO
STRI	286 (67.1%)	638 (69.0%)	446 (36.4%)
HOMO	132 (31.0%)	246 (26.6%)	756 (61.8%)
None	8 (1.9%)	23 (2.5%)	22 (1.8%)
total	426	925	1224

**Table 4.** Performance on proteins in STRI and HOMO

Method	F${}_{\text{max}}$			AUPR		
	MFO	BPO	CCO	MFO	BPO	CCO
STRI						
BLAST-KNN	0.608	0.291	0.570	0.466	0.122	0.438
LR-InterPro	0.630	0.293	0.627	0.562	0.162	0.598
Net-KNN	0.443	0.314	0.617	0.297	0.177	0.607
DeepGOCNN	0.432	0.258	0.588	0.173	0.036	0.136
DeepGOPlus	0.602	0.306	0.617	0.423	0.118	0.489
DeepGraphGO	**0.642**	**0.348**	**0.665**	**0.582**	**0.209**	**0.663**
HOMO						
BLAST-KNN	0.583	0.248	0.704	0.456	0.104	0.652
LR-InterPro	0.602	0.256	0.689	**0.501**	0.114	0.720
Net-KNN	0.422	0.300	0.709	0.253	0.128	0.675
DeepGOCNN	0.456	0.231	0.662	0.349	0.088	0.613
DeepGOPlus	0.582	0.257	0.710	0.438	0.100	0.656
DeepGraphGO	**0.619**	**0.306**	**0.726**	0.475	**0.157**	**0.736**

#### 4.5.3 Species (HUMAN and MOUSE) specific performance

We explored the performance of each species listed in Table 1, particularly HUMAN and MOUSE. Table 5 reports the performance of DeepGraphGO and competing methods over proteins in HUMAN and MOUSE. Again DeepGraphGO outperformed all competing methods in all twelve settings except one. DeepGraphGO has a notable feature, multispecies strategy, which uses proteins of all species in a single model at once. To understand the advantage of this feature of using the STRING network of all 17 species in the training set at once, we considered two variants of DeepGraphGO: (i) DeepGraphGO${}_{\text{sp}}$: trained with proteins in the target species only, and (ii) DeepGraphGO${}_{∼sp}$: trained with proteins in 16 species other than the target species. Table 6 shows the performance of DeepGraphGO and the two variants over test proteins in HUMAN and MOUSE. DeepGraphGO achieved the best performance in nine out of all 12 settings. Meanwhile, even without using proteins in the target species for training, DeepGraphGO${}_{∼sp}$ was one of the two best methods in eight out of all 12 settings. In contrast, DeepGraphGO${}_{\text{sp}}$ is generally the third best model, which was one of the two best methods in only five out of all 12 settings. For example, DeepGraphGO achieved the highest F${}_{\text{max}}$ of 0.638 on CCO over MOUSE proteins, which was followed by DeepGraphGO${}_{∼sp}$ (0.622) and DeepGraphGO${}_{\text{sp}}$ (0.602). All these results indicate that multispecies strategy by DeepGraphGO is helpful for solving AFP, allowing DeepGraphGO to outperform all other competing methods. In addition, the relatively good performance of DeepGraphGO${}_{∼sp}$ highlights (i) the importance of using more data than the target species and also (ii) the effectiveness of GCN of using such a large amount of data for AFP, which eventually allows to integrate both sequence/domain/family and network information.

**Table 5.** Performance comparison on proteins in HUMAN and MOUSE

Method	F${}_{\text{max}}$			AUPR		
	MFO	BPO	CCO	MFO	BPO	CCO
HUMAN (9606)						
BLAST-KNN	0.471	0.241	0.555	0.296	0.074	0.384
LR-InterPro	0.593	0.282	0.650	0.496	0.138	0.603
Net-KNN	0.485	0.261	0.615	0.358	0.143	0.620
DeepGOCNN	0.468	0.263	0.594	0.327	0.114	0.552
DeepGOPlus	0.501	0.277	0.625	0.246	0.088	0.479
DeepGraphGO	**0.633**	**0.320**	**0.655**	**0.520**	**0.178**	**0.642**
MOUSE (10090)						
BLAST-KNN	**0.681**	0.289	0.593	0.593	0.105	0.441
LR-InterPro	0.628	0.312	0.592	0.625	0.175	0.569
Net-KNN	0.420	0.302	0.588	0.319	0.167	0.569
DeepGOCNN	0.475	0.258	0.574	0.405	0.129	0.495
DeepGOPlus	0.634	0.306	0.598	0.550	0.132	0.488
DeepGraphGO	0.650	**0.329**	**0.638**	**0.651**	**0.201**	**0.634**

**Table 6.** Performance comparison of DeepGraphGO and the two variants over proteins in HUMAN and MOUSE

Method	F${}_{\text{max}}$			AUPR		
	MFO	BPO	CCO	MFO	BPO	CCO
HUMAN (9606)						
DeepGraphGO${}_{\text{sp}}$	**0.636**	0.299	0.629	**0.530**	0.163	0.601
DeepGraphGO${}_{∼sp}$	0.612	0.297	0.643	0.524	0.169	0.607
DeepGraphGO	0.633	**0.320**	**0.655**	0.520	**0.178**	**0.642**
MOUSE (10090)						
DeepGraphGO${}_{\text{sp}}$	0.559	0.309	0.602	0.499	0.183	0.584
DeepGraphGO${}_{∼sp}$	**0.653**	0.302	0.622	0.635	0.178	0.618
DeepGraphGO	0.650	**0.329**	**0.638**	**0.651**	**0.201**	**0.634**

#### 4.5.4 Performance comparison on *difficult* proteins

Inspired by the result analysis of CAFA2 (Jiang *et al.*, 2016), we examined the performance of competing AFP methods over *difficult* proteins, where the definition of the difficult proteins is: the sequence identity of the protein (in the training set) most similar (homologous) to a difficult protein is less than 60%. The number of *difficult* proteins in testing set is 303 in MFO, 649 in BPO and 437 in CCO, respectively. Note that obviously it is hard to make accurate function prediction of these *difficult* proteins by homology-based methods. Table 7 shows the performance comparison of DeepGraphGO and competing methods. DeepGraphGO achieved the best performance in all six settings, and LR-InterPro achieved the second best performance in five out of all six settings. For example, DeepGraphGO achieved the highest AUPR of 0.184, which was followed by LR-InterPro (0.148) and Net-KNN (0.142). LR-InterPro uses protein domain and family information, which made LR-InterPro outperform BLAST-KNN (in all six settings), which is a sequence homology-based method. We also found that the performance of DeepGOPlus was worse than LR-InterPro in five out of all six settings. A possible reason of this result would be that one of the two components of DeepGOPlus, DiamondScore, which is a homology-based method, did not work well for *difficult* proteins. On the other hand, by taking advantage of both protein domain/family and network information through GCN layers, DeepGraphGO could outperform LR-InterPro in all six settings. All these results suggest that DeepGraphGO is the most reliable and effective model among all compared methods for the AFP of *difficult* proteins.

**Table 7.** Performance comparison on difficult proteins

Method	F${}_{\text{max}}$			AUPR		
	MFO	BPO	CCO	MFO	BPO	CCO
BLAST-KNN	0.534	0.274	0.521	0.377	0.114	0.354
LR-InterPro	0.589	0.275	0.613	0.493	0.148	0.591
Net-KNN	0.404	0.292	0.595	0.230	0.142	0.560
DeepGOCNN	0.406	0.243	0.578	0.246	0.091	0.478
DeepGOPlus	0.564	0.292	0.602	0.326	0.108	0.454
DeepGraphGO	**0.598**	**0.322**	**0.625**	**0.508**	**0.184**	**0.607**

### 4.6 Results analysis

#### 4.6.1 Comparison over groups divided by #annotations per GO term

According to the number of annotations per GO term: we grouped annotations (GO terms) in the testing set into four groups: 10–30, 31–100, 101–300 and >300. Table 8 shows M-AUPR computed in each group. DeepGraphGO outperformed other methods in all 12 settings except for two cases, being followed by LR-InterPro and BLAST-KNN in MFO and CCO, respectively (each being one of the two best in three out of four cases). Deep learning-based methods showed the worst performance, particularly for less frequent GO terms. For example, DeepGOCNN showed only 0.014, 0.005 and 0.004 for the 10–30 group in MFP, BPO and CCO, respectively (The corresponding M-AUPR of DeepGraphGO was 0.594, 0.170 and 0.353, respectively).

**Table 8.** M-AUPR of DeepGraphGO and competing methods

Method	MFO				BPO				CCO			
	10–30	31–100	101–300	>300	10–30	31–100	101–300	>300	10–30	31–100	101–300	>300
BLAST-KNN	0.590	0.579	0.533	0.500	0.064	0.192	**0.141**	0.135	0.296	0.501	0.236	0.251
LR-InterPro	0.544	**0.652**	0.560	0.545	0.146	0.154	0.120	0.128	0.319	0.461	0.192	0.220
Net-KNN	0.281	0.371	0.301	0.273	0.034	0.131	0.123	0.144	0.126	0.258	0.167	0.248
DeepGOCNN	0.014	0.045	0.235	0.252	0.005	0.019	0.042	0.073	0.004	0.004	0.061	0.169
DeepGOPlus	0.309	0.322	0.414	0.427	0.022	0.078	0.096	0.124	0.170	0.418	0.193	0.239
DeepGraphGO	**0.594**	0.632	**0.571**	**0.575**	**0.170**	**0.196**	0.134	**0.168**	**0.353**	**0.559**	**0.277**	**0.295**

#### 4.6.2 Term-centric and pair-centric comparison with network-based methods over specific species (HUMAN and MOUSE)

Existing network-based methods focus more on the term-centric metric over a specific species, and so we compared DeepGraphGO with GeneMANIA, clusDCA and deepNF (state-of-the-art network-based methods) over HUMAN and MOUSE in term-centric and pair-centric manners. As the number of testing proteins in each species is limited, we collected all GO terms (appearing more than twice in the testing set) together to compute M-AUPR (and also regular AUPR was computed). Table 9 reports performance of DeepGraphGO and the three competing methods. DeepGraphGO achieved the best performance in all 12 settings, except two cases. For example, DeepGraphGO achieved the highest M-AUPR of 0.254, followed by GeneMANIA (0.203) and deepNF (0.148).

**Table 9.** M-AUPR and AUPR of DeepGraphGO and three network-based competing methods over proteins in HUMAN and MOUSE

	M-AUPR			AUPR		
	MFO	BPO	CCO	MFO	BPO	CCO
HUMAN (9606)						
GeneMAINIA	0.560	0.203	0.413	0.324	0.147	0.618
clusDCA	0.317	0.145	0.263	0.159	0.059	0.323
deepNF	0.600	0.148	**0.445**	0.476	0.148	**0.654**
DeepGraphGO	**0.672**	**0.254**	0.362	**0.520**	**0.178**	0.642
MOUSE (10090)						
GeneMAINIA	0.514	0.192	0.487	0.230	0.154	0.511
clusDCA	0.465	0.167	0.397	0.217	0.095	0.383
deepNF	0.603	0.227	0.476	0.387	0.152	0.588
DeepGraphGO	**0.770**	**0.244**	**0.547**	**0.651**	**0.201**	**0.634**

#### 4.6.3 Integrating DeepGraphGO as a component method of NetGO

Table 10 shows the performance of DeepGraphGO, two state-of-the-art ensemble methods for AFP, GOLabeler and NetGO and DeepGraph-LTR, which is generated by plugging DeepGraphGO into NetGO as a component to improve the performance. From Table 10, we have two findings: (i) DeepGraphGO (again which uses both network and protein domain/family information) outperformed GOLabeler (which does not use network information) in both BP and CC in terms of F${}_{\text{max}}$, while the performance of DeepGraphGO was slightly worse than NetGO, the state-of-the-art method. (ii) DeepGraphGO-LTR achieved the best performance in all six cases. For example, the highest AUPR of 0.202 in BPO was 6% higher than NetGO (0.190) and 35.6% higher than GOLabeler (0.149). Overall, the AFP performance could be further improved in all three GO domains by using DeepGraphGO as a component of NetGO.

**Table 10.** Performance comparison of DeepGraphGO and ensemble methods over the whole testing set

Method	F${}_{\text{max}}$			AUPR		
	MFO	BPO	CCO	MFO	BPO	CCO
DeepGraphGO	0.623	0.327	0.692	0.543	0.194	0.695
GOLabeler	0.629	0.296	0.685	0.558	0.149	0.708
NetGO	0.630	0.335	0.697	0.553	0.190	0.725
DeepGraphGO-LTR	**0.634**	**0.339**	**0.702**	**0.574**	**0.202**	**0.736**

#### 4.6.4 Ablation experiment on GCN layers with protein network

The main feature of DeepGraphGO is the GCN layers for the input protein network. Instead of the GCN layer, we train representation vectors by using a fully connected layer for the input InterPro feature vectors. We call this alternative as DNN-InterPro. Table 11 reports the performance of DeepGraphGO and DNN-InterPro. We found that DeepGraphGO outperformed DNN-InterPro in all six cases with all three domains. For example, DeepGraphGO achieved 0.327 of F${}_{\text{max}}$ and 0.194 of AUPR in BPO, which were 15.1% and 22.8%, respectively, higher than DNN-InterPro. This result indicates again that the GCN layer in DeepGraphGO is effective for improving the performance of AFP.

**Table 11.** Performance comparison of DeepGraphGO and DNN-InterPro

Method	F${}_{\text{max}}$			AUPR		
	MFO	BPO	CCO	MFO	BPO	CCO
DNN-InterPro	0.607	0.284	0.663	0.513	0.158	0.667
DeepGraphGO	**0.623**	**0.327**	**0.692**	**0.543**	**0.194**	**0.695**

#### 4.6.5 Case study

Finally we show a typical example obtained by DeepGraphGO and competing methods, to illustrate the real performance difference on annotating GO to a *no-knowledge* protein in the testing set. Table 12 shows the GO terms in BPO for the target *no-knowledge* protein, Q9BQD7, which were predicted by competing methods and DeepGraphGO. In Table 12, the bottom row shows 22 true GO terms of Q9BQD7, and in each row, correctly predicted GO terms were in red. Q9BQD7 is a *difficult* protein, which has no homologous proteins (cut-off e-value at 0.001) in the training set, by which BLAST-KNN could not predict any GO terms. LR-InterPro predicted 12 true GO terms out of the predicted 19 GO terms. Net-KNN predicted the largest number (45) of GO terms, out of which 18 was true. DeepGOCNN predicted 20 GO terms, with eight true GO terms, while DeepGOPlus predicted only five GO terms, with four true GO terms. This may be due to that one homology-based component, DiamondScore, did not work well on the *difficult* protein. Finally DeepGraphGO achieved 18 true GO terms out of 25 predicted. Thus 18 was the highest number of correctly predicted GO terms (by DeepGraphGO and NetKNN), while the number of wrongly predicted GO terms was only 7 by DeepGraphGO and 23 by Net-KNN. This difference was clearly shown by the difference in the F1 score in the last column. That is, DeepGraphGo achieved 0.766 of F1 while Net-KNN was 0.537, which was even lower than 0.585 of LR-Interpro. Supplementary Figure S1 in Supplementary Materials shows the DAG with these 22 GO terms, where each GO term is attached with the methods, which predict the corresponding GO term correctly. Overall this real case study demonstrates the high predictive performance of DeepGraphGO over other competing methods.

**Table 12.** Predicted GO terms (the root GO term (GO:0008150 biological process) is omitted) of Q9BQD7 in BPO by NetGO and competing methods

Method		F1
BLAST-KNN		0.0
LR-InterPro	GO:0006139, GO:0006725, **GO:0006807**, **GO:0008152**, **GO:0009987**, **GO:0032259**, GO:0034641, **GO:0043170**	0.585
	**GO:0043412**, **GO:0043414**, **GO:0044237**, **GO:0044238**, **GO:0044260**, GO:0046483, GO:0065007, **GO:0071704**	
	GO:0090304, GO:1901360, **GO:1901564**	
Net-KNN	GO:0006139, GO:0006412, **GO:0006464**, **GO:0006479**, GO:0006518, GO:0006725, **GO:0006807**, **GO:0008152**	0.537
	**GO:0008213**, GO:0009058, GO:0009059, **GO:0009987**, GO:0010467, GO:0010468, GO:0016070, GO:0019222	
	**GO:0019538**, **GO:0032259**, GO:0034641, GO:0034645, **GO:0036211**, GO:0043043, **GO:0043170**, **GO: 0043412**	
	**GO:0043414**, GO:0043603, GO:0043604, **GO:0044237 GO:0044238**, GO:0044249, **GO:0044260**, **GO:0044267**	
	GO:0044271, GO:0046483, GO:0048519, GO:0050789, GO:0050794, GO:0060255, GO:0065007, **GO:0071704**	
	GO:0090304, GO:1901360, **GO:1901564**, GO:1901566, GO:1901576	
DeepGOCNN	**GO:0044238**, **GO:1901564**, **GO:0008152**, **GO:0043170**, **GO:0044237**, **GO:0006807**, **GO:0009987**, **GO:0071704**	0.381
	GO:0050896, GO:0050794, GO:0050789, GO:0031323, GO:0048519, GO:0065007, GO:0019222, GO:0080090	
	GO:0060255, GO:1901576, GO:0009058, GO:0044249	
DeepGOPlus	**GO:0009987**, **GO:0008152**, **GO : 0071704**, **GO:0044237**, GO:0065007	0.296
DeepGraphGO	**GO:0006464**, **GO:0006479**, **GO:0006807**, **GO:0008152**, **GO:0008213**, GO:0009058, **GO:0009987**, **GO:0019538**	**0.766**
	**GO:0032259**, GO:0034641, **GO:0036211**, **GO:0043170**, **GO:0043412**, **GO:0043414**, **GO:0044237**, **GO:0044238**	
	GO:0044249, **GO:0044260**, **GO:0044267**, GO:0050789, GO:0065007, **GO:0071704**, GO:0071840, **GO:1901564**	
	GO:1901576	
Truth	GO:0044238, GO:0006479, GO:0032259, GO:0044237, GO:0018193, GO:0036211, GO:0008152, GO:0009987,	
	GO:0008213, GO:0043414, GO:0043412, GO:0006807, GO:0018205, GO:0006464, GO:0044267, GO:0071704,	
	GO:0019538, GO:1901564, GO:0044260, GO:0043170, GO:0018022, GO:0018023	

*Note*: Correctly predicted GO terms are in red. The last column shows F1 scores.

## 5 Conclusion

We have designed an end-to-end, graph neural network-based model, DeepGraphGO, for the challenging AFP problem, to make the most of both protein sequence and protein network information. DeepGraphGO uses ‘multispecies strategy’, which allows only one single model to be trained by using proteins of all species. Extensive experiments under diverse settings revealed that DeepGraphGO outperformed a number of compared methods, such as DeepGOCNN, DeepGOPlus and three representative network-based methods, GeneMANIA, deepNF and clusDCA. Furthermore DeepGraphGO can be integrated into an ensemble method as a component. Then DeepGraphGO-LTR, a method obtained by plugging DeepGraphGO into NetGO, the state-of-the-art ensemble method of AFP, outperformed both GOLabeler and NetGO. Possible future work would be to build a single model for AFP, which can incorporate all kind of protein information including sequence, structure and network.

*Financial Support*: S.Z. was supported by National Natural Science Foundation of China (No. 61872094), Shanghai Municipal Science and Technology Major Project (No.2018SHZDZX01), ZJ Lab, and Shanghai Center for BrainScience and Brain-Inspired Technology. S.Y. and R.Y. have been supported by the 111 Project (No. B18015), Shanghai Municipal Science and Technology Major Project (No. 2017SHZDZX01) and Information Technology Facility, CAS-MPG Partner Institute for Computational Biology, Shanghai Institute for Biological Sciences, Chinese Academy of Sciences.

H. M. has been supported partially by Academy of Finland (315896), JST ACCEL (JPMJAC1503), NEXT KAKENHI (19H04169).

*Conflict of Interest*: none declared.

## Supplementary Material

btab270_Supplementary_Data
