# Supplementary material for: DeepGraphGO: graph neural network for large-scale, multispecies protein function prediction
Source: Bioinformatics. 2021 Jul 12;37(Suppl 1):i262–71. doi: 10.1093/bioinformatics/btab270 (PMC8294856; doi:10.1093/bioinformatics/btab270)
Supplement: btab270_Supplementary_Data [file btab270_supplementary_data.pdf]

Supplementary Material of DeepGraphGO

Ronghui You[1], Shuwei Yao[1], Hiroshi Mamitsuka[2,3], and Shanfeng Zhu[4,5,6,*]

[1]School of Computer Science, Fudan University, Shanghai 200433, China.
[2]Bioinformatics Center, Institute for Chemical Research, Kyoto University, Uji, Kyoto Prefecture, Japan.
[3]Department of Computer Science, Aalto University, Espoo, Finland.
[4]Institute of Science and Technology for Brain-Inspired Intelligence and Shanghai Institute of Artificial Intelligence Algorithms, Fudan University, Shanghai 200433, China.
[5]Ministry of Education, Key Laboratory of Computational Neuroscience and Brain-Inspired Intelligence (Fudan University), China.
[6]Shanghai Key Lab of Intelligent Information Processing, Fudan University, Shanghai 200433, China.

1 Result

Table 1 reports the average performance of DeepGraphGO and other competing methods using 100 bootstrapped datasets with replacement. We use a paired *t*-test to statistically evaluate the performance difference between the best method (in boldface in tables) and all other methods. If *p*-value is smaller than 0.05, the improvement is considered as statistically significant. Table 2 reports the performance (F_{max}) and training time (hours) of DeepGraphGO with different M(number of GCN layer) settings by a single NVIDIA V100 GPU. From the results we can see that the performance was only slightly improved regardless of the dramatically increase of computational cost. Then we set $M = 2$.

Table 1: The performance comparison of DeepGraphGO and other competing methods using 100 bootstrapped datasets with replacement.

Method	F_{max}			AUPR		
	MFO	BPO	CCO	MFO	BPO	CCO
BLAST-KNN	0.592	0.274	0.652	0.458	0.114	0.572
	5.22e-52	1.49e-92	9.14e-87	8.68e-76	6.36e-100	3.98e-112
LR-InterPro	0.617	0.280	0.661	0.532	0.145	0.671
	3.04e-14	1.91e-96	6.53e-85	8.11e-20	1.80e-87	5.71e-49
Net-KNN	0.425	0.306	0.667	0.274	0.157	0.642
	7.94e-116	1.57e-59	2.05e-75	2.93e-111	1.02e-66	2.47e-80
DeepGOCNN	0.436	0.248	0.633	0.309	0.102	0.573
	2.30e-111	1.02e-106	1.24e-103	2.46e-108	2.56e-99	1.01e-113
DeepGOPlus	0.597	0.291	0.674	0.402	0.110	0.596
	5.15e-49	1.40e-77	2.14e-57	1.55e-97	4.63e-104	3.48e-108
DeepGraphGO	**0.624**	**0.327**	**0.692**	**0.545**	**0.195**	**0.695**

Table 2: F_{max} and training time (hours) with different settings of M(number of GCN layers).

M	F_{max}			AUPR			Training Time (H)		
	MFO	BPO	CCO	MFO	BPO	CCO	MFO	BPO	CCO
$M = 2$	0.623	0.327	0.692	0.543	0.194	0.695	2.7	4.5	3.4
$M = 3$	**0.625**	0.333	0.693	**0.549**	0.204	**0.699**	40.2	69.0	48.5
$M = 4$	0.621	**0.334**	**0.694**	0.541	**0.212**	0.696	**109.25**	**186.05**	**144.8**

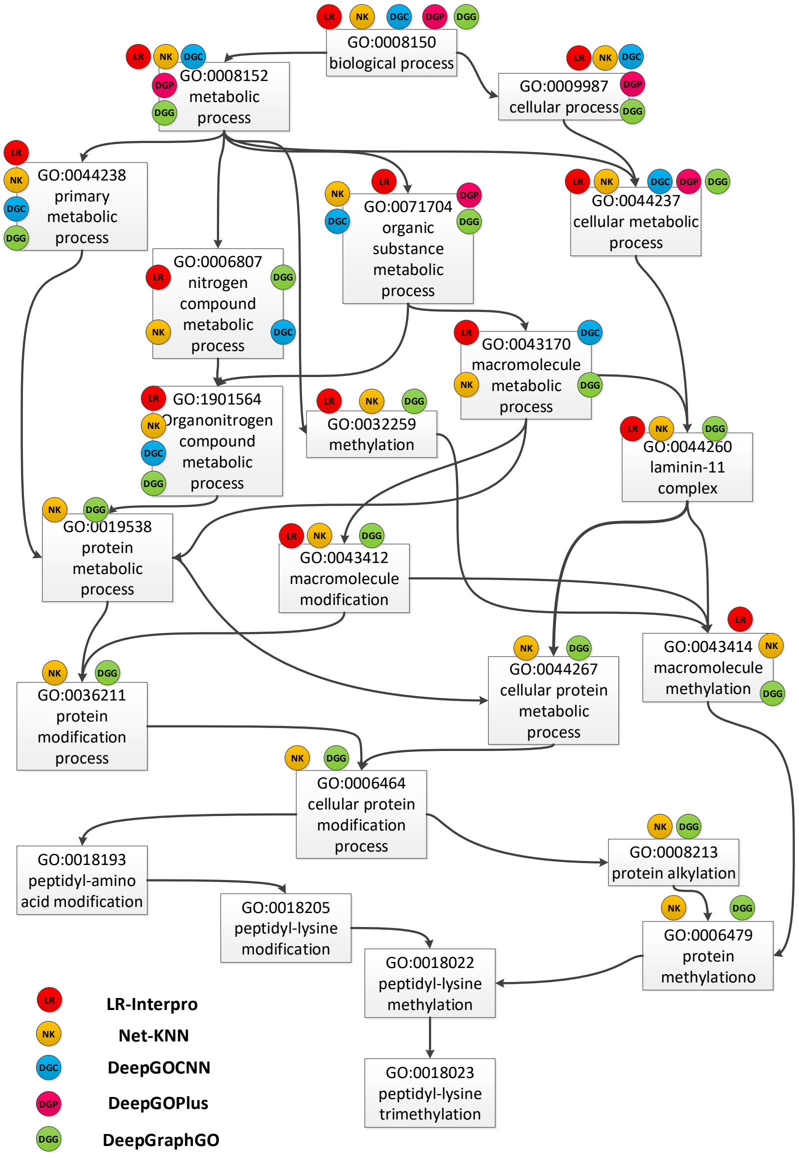

Figure 1: GO terms of Q9BQD7 in the DAG of BPO and the methods, which correctly predicted the corresponding GO term.
